# Human bocavirus 1 is a genuine pathogen for acute respiratory tract infection in pediatric patients determined by nucleic acid, antigen, and serology tests

**DOI:** 10.3389/fmicb.2022.932858

**Published:** 2022-07-29

**Authors:** Ri De, Ke-Xiang Zhang, Fang Wang, Yu-Tong Zhou, Yu Sun, Dong-Mei Chen, Ru-Nan Zhu, Qi Guo, Shuang Liu, Dong Qu, Yuan Qian, Lin-Qing Zhao

**Affiliations:** ^1^Laboratory of Virology, Beijing Key Laboratory of Etiology of Viral Diseases in Children, Capital Institute of Pediatrics, Beijing, China; ^2^Graduate School of Peking Union Medical College, Beijing, China; ^3^Department of Intensive Care Unit, Affiliated Children's Hospital, Capital Institute of Pediatrics, Beijing, China

**Keywords:** human bocavirus 1, pediatric patients, pathogenic role, acute respiratory tract infection (ARTI), nasopharyngeal aspirates

## Abstract

**Background:**

Human bocavirus 1 (HBoV1), first discovered in 2005, was positive in symptomatic and healthy children and co-detected with other respiratory viruses. It is a long journey to decisively demonstrate the unique viral pathogenic function of acute respiratory tract infection (ARTI) in pediatric patients.

**Methods:**

Respiratory specimens collected from pediatric patients with ARTI from January 2017 to December 2021 were screened by a capillary electrophoresis-based multiplex PCR (CEMP) assay, then genotyped by PCR and sequencing for HBoV1. For the antigen test, a part of HBoV1 DNA positive nasopharyngeal aspirates (NPAs) was used as an antigen, while a rabbit anti-HBoV1 DR2 specific to HBoV1 was used as an antibody in the indirect-immunofluorescence assay (IFA). Finally, the levels of IgG specific to HBoV1 in acute and convalescent sera selected retrospectively from only HBoV1 DNA-positive patients were evaluated by IFA.

**Results:**

Among 9,899 specimens, 681 were positive for HBoV1 DNA (6.88%, 681/9899), which included 336 positives only for HBoV1 (49.34%, 336/681) and 345 (50.66%, 345/681) positives also for other pathogens. In the antigen test, there were 37 among 47 NPAs determined as HBoV1 antigen-positive (78.72%, 37/47), including 18 (48.65%, 18/37) positives solely for HBoV1 DNA. Among 4 pediatric patients with both acute and convalescent sera, there was one positive for HBoV1 antigen (D8873) and 2 lack the antigen results (D1474 and D10792), which showed seroconversion with a ≥ 4-fold increase in IgG levels.

**Conclusions:**

The combination results of nucleic acid, antigen, and serology tests answered that HBoV1 is a genuine pathogen for ARTI in pediatric patients.

## Keypoints

- Among 9,899 pediatric patients with acute respiratory tract infection (ARTI), 681 (6.88%) were positive for human bocavirus 1 (HBoV1) DNA.
- There were 37 patients who were positive for HBoV1 antigen among 47 tested.
- One pediatric patient was positive for HBoV1 DNA, antigen, and seroconversion in IgG levels.

## Introduction

Human bocavirus (HBoV) was first reported in 2005 by Allander et al. in nasopharyngeal aspirates (NPAs) from children with acute respiratory tract infection (ARTI) through metagenomic detection systems for active virus hunting (Allander et al., 2005). This HBoV was then named HBoV1 to distinguish it from HBoV2, 3, and 4, similarly detected in 2008–2010 in human stools searching for novel viruses causing gastroenteritis (Kapoor et al., 2009, 2010).

Soon after HBoV1's discovery, many studies reported the presence of its DNA in symptomatic children, suggesting its relationship with acute respiratory infections (Bastien et al., 2006; Zhao et al., 2006; Simon et al., 2007). However, the clinically relevant diagnosis of HBoV1 is challenging, as the virus is frequently detected in healthy children (García-García et al., 2008) and co-findings with other respiratory viruses are common. In addition to stool and nasopharyngeal aspirates, the viruses have been found in serum and cerebrospinal fluids and sewage and river waters (Zhao et al., 2014; Kumthip et al., 2021). Therefore, many researchers thought that HBoV1 was just an innocent bystander not causing illness. To demonstrate its pathogenic role conclusively is a long journey full of potential pitfalls (Jartti et al., 2012; Söderlund-Venermo, 2019).

Koch's postulates are a series of ground rules to determine the causal relationship between microbes and hosts (Sakula, 1983; Li D. et al., 2019; Jain et al., 2021). However, it is not easy for those microbes without susceptible cells to apply Koch's postulates to explain the causal relationships between hosts and microbes (Hosainzadegan et al., 2020).

It has been suggested that a high viral load of over 10^4−6^ copies/ml in the nasopharyngeal specimens (NPS) determined by quantitative PCR (qPCR) gives a higher probability for the current infection to be caused by HBoV1. However, as confirmed by serology, one-third of the patients with a low viral load and even a few with HBoV1-PCR-negative NPS suffered from a genuine acute HBoV1 infection, rendering qPCR inaccurate (Söderlund-Venermo et al., 2009). Serological studies used recombinant HBoV1 capsid proteins as antigens to build the causal relationships between ARTI-HBoV1 based on a seroconversion or a ≥4-fold increase in IgG levels, which could not rule out cross-reactivity with the capsid proteins of the other three HBoV species and the original antigenic sin (OAS) phenomenon (Kantola et al., 2008; Li et al., 2015). In a pediatric patient with respiratory tract infection symptoms, capsid proteins can be detected in NPA by a commercial rapid antigen-detection assay (Bruning et al., 2016).

Combining techniques are preferred to determine an accurate pathogen due to all of the drawbacks outlined for each method (Jartti et al., 2012). In the present study, based on these combining results of nucleic acid, antigen, and antibody detection, the pathogenic role of HBoV1 in ARTI among pediatric patients was built.

## Materials and methods

### Clinical specimens

From January 2017 to December 2021, clinical specimens, such as throat swabs, nasopharyngeal swabs, nasopharyngeal aspirates (NPAs), and bronchoalveolar lavage fluids, were collected from pediatric patients with ARTI visiting or admitted to the Affiliated Children's Hospital, Capital Institute of Pediatrics for respiratory pathogen screening using a capillary electrophoresis-based multiplex PCR (CEMP)-compatible assay-Respiratory Pathogen Multiplex Detection Kit (Ningbo HEALTH Gene Technologies Ltd., Ningbo, China) (Li X. et al., 2019).

Clinical specimens were centrifuged at 500 g for 10 min upon arrival at the laboratory. The supernatants were used for nucleic acid extraction, and the leftovers were stored at −80°C for further use. The cell pellets from a part of NPAs positive for HBoV were resuspended and spotted onto slides. The slides were acetone fixed and kept at −20°C, which would be used as an antigen in IFA.

### Nucleic acid extraction

Nucleic acid was extracted from 140 μl of each specimen using the QIAamp® Viral RNA Mini Kit (250) (Qiagen, Germany) according to the manufacturer's instructions.

### CEMP assay for clinical specimens

According to the manufacturer's instructions, 15 pairs of primers for detecting 13 pathogens, deoxynucleoside triphosphates (dNTPs), MgCl_2_, and buffer were included in the kit for multiplex PCRs. Then, the amplification products were subjected to capillary electrophoresis on a GeXP capillary electrophoresis system (Sciex, Concord, ON, Canada) to detect the signals of the 15 labeled PCR products measured by fluorescence and separated by size: influenza virus (Flu) A 105 nt (2009H1N1 163.3 nt, H3N2 244.9 nt), FluB 212.7 nt, human adenovirus virus (HAdV) 110.2/113.9 nt (representing different subtypes), human bocavirus (HBoV) 121.6 nt, human rhinovirus (HRV) 129.6 nt, human parainfluenza virus (PIV) 181.6 nt, chlamydia (Ch) 190.5 nt, human metapneumovirus (HMPV) 202.8 nt, mycoplasma (Mp) 217 nt, human coronavirus (HCoV) 265.1 nt, and respiratory syncytial virus (RSV) 280.3 nt. The peak value of HBoV in the amplification product shown in the study is an indirect signal marker of the number of nucleotides in the template, which were then converted to the viral load (copies/ml) (Li X. et al., 2019).

### Genotyping of HBoVs by PCR

For HBoV genotyping, HBoV positive samples were used to amplify the 690-nt fragment at the NP1 and VP1 gene boundaries using primers HBoV-c1 (5′-CTTYGAA GAYCTCAG ACC-3′) and HBoV-c2 (5′-TKGAKCCAA TAATKCC AC-3′), followed by sequencing and phylogenetic analysis as described previously (Zhao et al., 2014).

### Harvesting HBoV1 VP3 expressed in HEK293 cells as antigen

The coding regions of the HBoV1 VP3 gene (nt 3,443–5,071 of ABK32030.1) (Schildgen et al., 2008) were inserted into the eukaryotic expression vector pcDNA3.1 (Invitrogen, USA) and transfected into HEK293 cells using Lipofectamine® 3000, P3000TM reagents, and Opti-MEM® (Invitrogen Life Technologies) according to the protocol manual. At 48 h of post-transfections, the HEK293 cells were harvested and centrifuged at 4,000 rpm for 5 min. Pellets were resuspended in phosphate-buffered saline (PBS) and spotted onto acetone-cleaned slides. The slides were acetone fixed and kept at −20°C, which would be used as an antigen in IFA.

### Antigen test by indirect-immunofluorescence assay

In IFA, in an assay based on home-brew rabbit serum obtained from the vaccination of rabbits with a short peptide (^195^IENELADLDGNAAGGNATEKALLYQM^220^) located on HBoV1 VP3 (GenBank accession No: ABK32030.1), the specificity of the rabbit anti-HBoV1 DR2 sera was tested by using slides of RSV, FluA and B, HAdV, PIV 1, 2, and 3 infected cells in the D3 Ultra™ DFA Respiratory Virus Screening and ID Kit (Diagnostic Hybrids Inc., Athens, OH, USA) as antigens, while its sensitivity was tested by using slides of HBoV1 VP3 expressed in HEK 293 cell as antigens.

The diluted anti-HBoV1 DR2 was added to the slides as antigens, then incubated in a moist box at 37°C for 30 min. After washing the slides three times in PBST, the slides were incubated with FITC-conjugated goat anti-rabbit IgG antibodies (1:1,000 dilution) (Zhongshan, China) at 37°C for 30 min. Furthermore, the slides were washed three times in PBST and were observed under the fluorescent microscope (Nikon Eclipse 80i, Nikon Corporation, Japan). Those green cells were positive for the specific HBoV1 antigen.

### Seroconversion test of IgG specific to HBoV1 by IFA

The acute and convalescent sera were retrospectively searched from the leftover sera for the clinical tests from pediatric patients with ARTI who were confirmed as HBoV1 positive in respiratory pathogen screening using CEMP assay. In IFA, by using anti-HBoV1 DR2 as a positive control, the acute sera, in-stock solution and 1:10 and 1:20 dilutions, and the convalescent sera in 1:10, 1:20, 1:80, and 1:160 dilutions were added to the positive control slides with purified VP3 of HBoV1 expressed or NPAs positive for HBoV1 DNA in CEMP assay. Incubated in a moist box at 37°C for 30 min and then washed three times in PBST, the slides were incubated with FITC-conjugated goat anti-human IgG antibodies (1:1,000 dilution) (Zhongshan, China) at 37°C for 30 min. The slides were washed three times in PBST and were observed under the fluorescent microscope. The sera with specific green cells were positive for HBoV1 IgG.

## Results

### Respiratory pathogen screening using CEMP assay

From January 2017 to December 2021, 10,306 clinical specimens were collected for respiratory pathogen screening using the CEMP assay. After removing duplicates during one hospitalization, 9,899 specimens from children of both sexes (boys vs. girls) at ratios of 1.45:1, with an average age of 3.235 ± 3.550 years, were included for further analysis, including 5,352 throat swabs, 2,485 nasopharyngeal swabs, 1,737 NPAs, and 325 bronchoalveolar lavage fluids. Among these, 681 were positive for HBoV (6.88%, 681/9899) (boys vs. girls, 1.70:1; average age, 2.345 ± 1.946 years), which were all confirmed as HBoV1 by genotyping PCR and sequence analysis, including 336 specimens only positive for HBoV1 (49.34%, 336/681) (boys vs. girls; 1.85:1, average age, 2.390 ± 1.851 years), and 345 specimens (50.66%, 345/681) also positive for other pathogens in which HRV (33.62%, 116/345) was the most common one, followed by RSV (14.49%, 50/345), MP (10.43%, 36/345), HPIV (8.41%, 29/345), HAdV (4.64%, 16/345), HMPV (4.35%, 15 /345), Flu (3.19%, 11/345), HCoV (1.45%, 5/345), and Ch (0.29%, 1/345), while there were 50 specimens positive for other two and 16 for other three pathogens.

**Figure 1:**
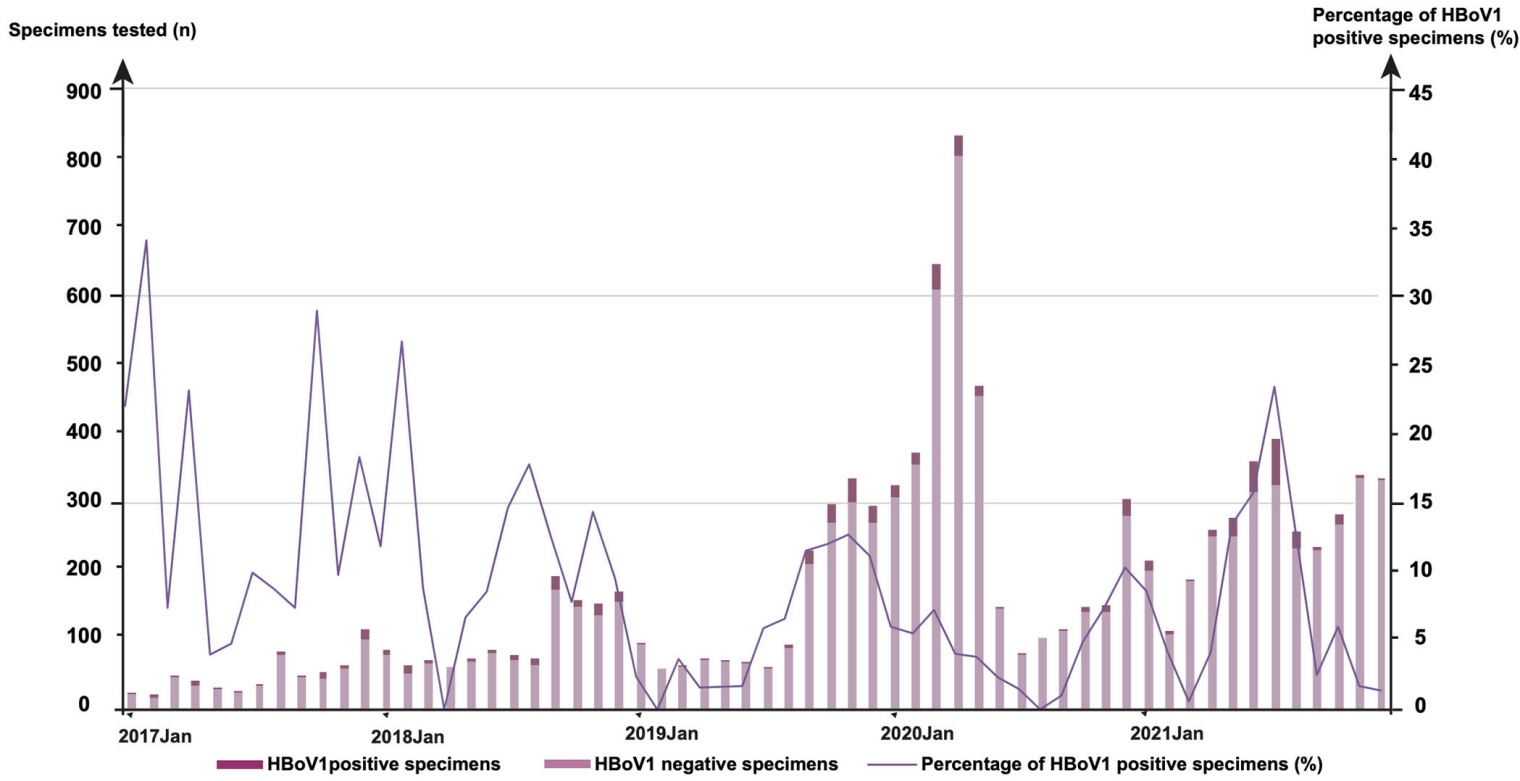
The monthly distribution of 681 human bocavirus 1 (HBoV1) positive specimens among 9,899 tested from January 2017 to December 2021.

The monthly distribution of 681 positives was observed for HBoV1 among 9,899 specimens (Figure 1). In 2017, the highest HBoV1 positive rate was shown in February (29.41%, 5/17), followed by that in October (25.00%, 11/44), April (20.00%, 7/35), January (19.05%, 4/21), and December (15.84%, 16/101). In 2018, the highest one is in February (23.08%, 12/52), followed by that in August (15.38%, 10/65), July (12.68%, 9/71), November (12.41%, 17/137), September (10.92%, 19/174), and January (10.26%, 8/78). In 2019, there were more HBoV1 positive specimens in November (10.96%, 33/301), October (10.37%, 28/270), and September (9.95%, 21/211). In 2020, the highest HBoV1 positive rate was shown in December (8.93%, 25/280); in 2021, the highest HBoV1 positive rate was shown in summer (in July, 20.25%, 66/326; in June, 13.61%, 43/316; in May, 11.60%, 29/250, and in Aug, 11.16%, 26/233).

### Specificity and sensitivity of anti-HBoV1 DR2 in antigen test by IFA

In the specificity test of anti-HBoV1 DR2 determined by IFA, no positive cell was captured in anti-HBoV1 DR2 sera reacting with control slides of RSV, Flu A and B, HAdV, and PIV 1-3 (Supplementary Figure S1).

In the sensitivity test of anti-HBoV1 DR2, specific green fluorescent cells (positive cells) were shown in the reaction of rabbit anti-HBoV1 DR2 sera till the dilution of 1:160 with HBoV1 VP3, as well as with clinical specimen D9466, while more positive cells and stronger fluorescent signal in anti-HBoV1 DR2 were shown in dilutions of 1:40 and 1:80 than that in the dilution 1:160 (Supplementary Figure S2).

### HBoV1 antigen test in NPAs from pediatric patients with ARTI by IFA

Among 681 HBoV1 nucleic acid positive specimens confirmed by the CEMP assay, pellets of 47 NPAs were spotted on slides for the HBoV1 antigen test by IFA with rabbit anti-HBoV1 DR2 sera diluted to 1:20 and 1:40 used as the antibody. These specimens were collected from 47 pediatric patients, including three who were 0–6 months old (6.38%, 3/47); 5 who were 6–12months old (10.64%, 5/47); 12 who were 1–2 years old (25.53%, 12/47); 14 who were 2–3 years old (29.79%, 14/47); 6, 3–4 years old (12.77%, 6/47); and 7 who were 4–5 years old (14.89%, 7/47).

**Figure 2:**
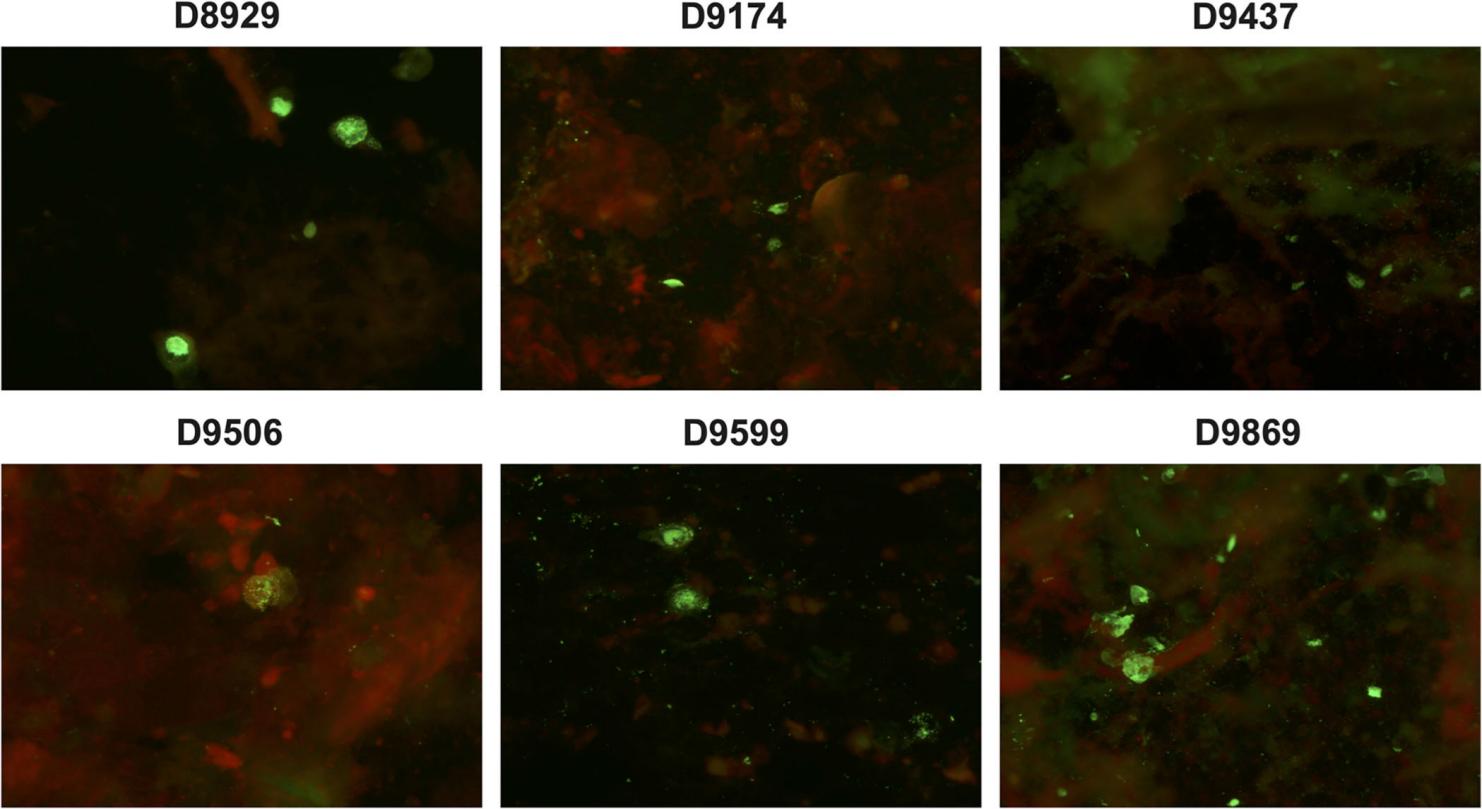
Specific green fluorescent cells are shown in the antigen test of clinical specimens D8929, D9174, D9437, D9506, D9599, and D9869 reacting with anti-HBoV1 DR2 by indirect-immunofluorescence assay (IFA).

Under the fluorescent microscope, specific positive green cells were observed in 37 NPAs (78.72%, 37/47) (Figure 2) with a mean viral load of 5.93 ± 1.48 × 10^4^ copies/ml of HBoV1 DNA in the CEMP assay and no positive cells in 10 NPAs (21.28%, 10/47) with a mean viral load of 5.64 ± 0.99 × 10^4^ copies/ml (*t* = 1.119, *p* = 0.269).

**Figure 3:**
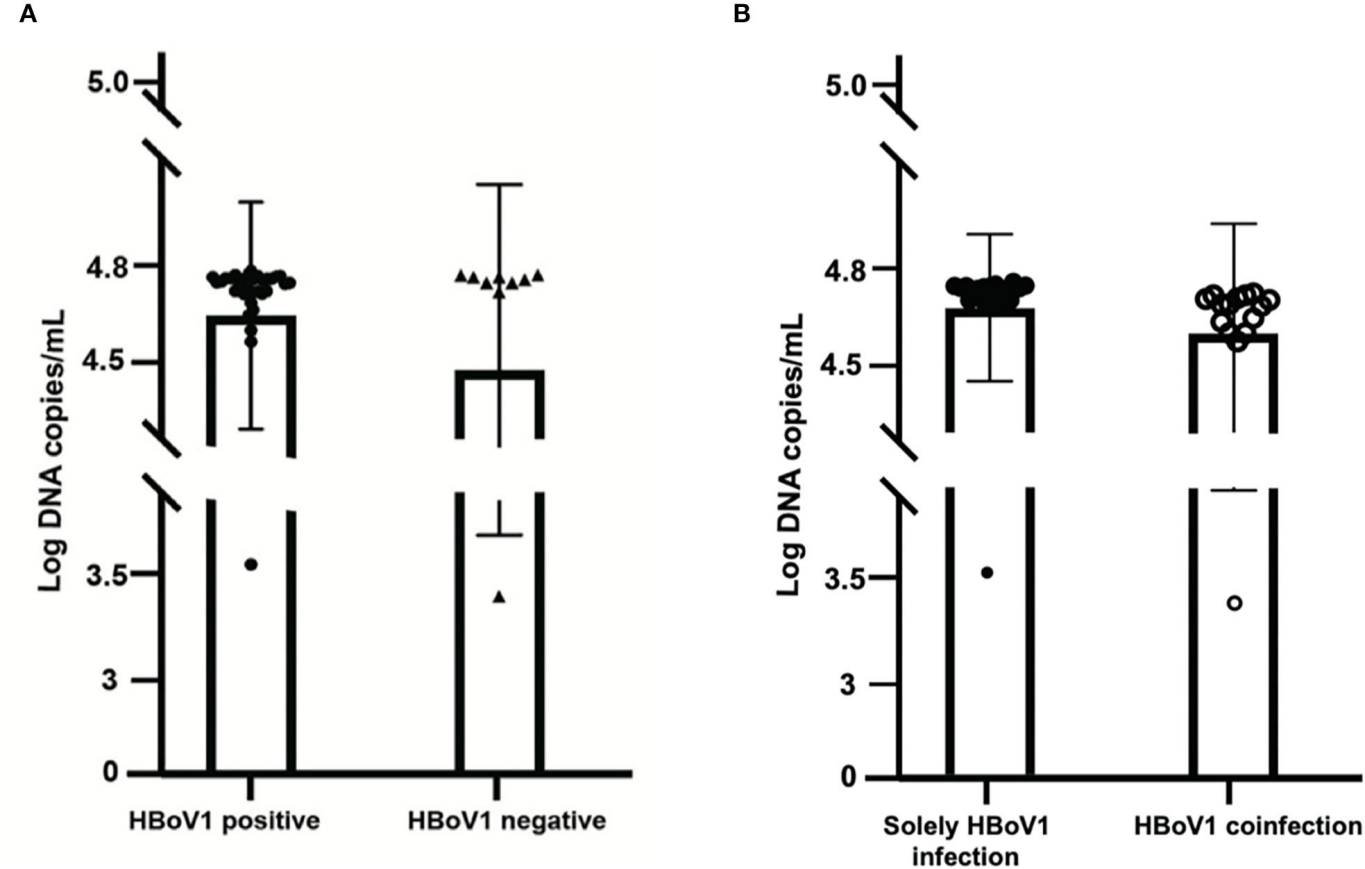
Comparison of the viral load of 37 specimens positive for the HBoV1 antigen in black spots with 10 specimens negative for HBoV1 antigen in black triangles **(A)**. The comparison of viral load in HBoV1 positive antigen in black spots with negative antigen specimens from pediatric patients with acute respiratory infection in black circles **(B)**.

Among these 37 HBoV1 antigen-positive specimens, 18 (48.65%, 18/37) were solely positive for HBoV1 DNA, while 19 (51.35%, 19/37) were also positive for other pathogens in the CEMP assay (Figure 3A). The mean viral load of those 18 solely HBoV1 nucleic acid-positive specimens was 59,846.7 ± 4,602.3 DNA copies/ml, while that of 19 positives for other pathogens was 58,286.5 ± 6,829.4 DNA copies/ml (*t* = 0.832, *p* = 0.411) (Figure 3B).

### Seroconversion of IgG specific to HBoV1 using HBoV1 VP3 or clinical specimens as antigen in IFA

By retrospective search, we found 4 pairs of acute and convalescent sera from patients with ARTI who were confirmed as solely HBoV1 DNA positive in the CEMP assay.

The first one was collected from a 4-year-old boy who suffered severe pneumonia and was admitted to the intensive care unit (ICU) on 22 June 2021. On the first day of the boy's admission, the NPA specimen D8873 and the acute serum CMV97128 were collected. After his discharge from our hospital on 29 June 2021, to improve his condition, his convalescent serum CMV97830 was collected when he revisited the hospital on 8 July 2021. The NPA specimen D8873 was HBoV1 positive, not only in the nucleic acid test (with a viral load of 5.98 × 10^4^ copies/ml) but also in the antigen test by IFA. Compared with the acute sera CMV97128 with negative IgG results in the stock solution, the convalescent CMV97830 showed positive IgG results until dilution 1:80.

The second one was collected from a 1-year-old boy diagnosed with acute asthmatic bronchitis and was admitted to the respiratory department on 21 September 2018. On his first day of admission, the NPA specimen D1474 and the acute serum CMV65471 were collected; then, his convalescent sera CMV65972 was collected on 10 October 2018, after his discharge on 28 September 2018. Although D1474 was only positive for HBoV1 DNA in the CEMP assay (6.05 × 10^4^ copies/ml), the HBoV1 antigen result was missed in IFA for the lack of cells on slides. CMV65972 was the first convalescent sera identified as positive for HBoV1 specific IgG, not only in reacting with VP3 but also with several clinical specimens, such as D9466.

The third one showed similar results to the second one. Its NPA specimen D10792, collected from a 3-year-old girl diagnosed with pneumonia on 27 January 2021, showed HBoV1 as the only positive one in the CEMP assay (5.15 × 10^4^ copies/ml), and the missed antigen results in IFA because of the full of squamous epithelial cells on slides. Her convalescent serum lcmv4945, collected on 5 January 2022, showed over 160-fold higher IgG titer than that of lcmv4565 collected on 27 December 2021.

The fourth one was from a 3-year-old boy diagnosed with pneumonia and admitted to the respiratory department on 29 June 2021. His NPA specimen D8954 collected on the admission day was only positive for HBoV1 by the CEMP assay (6.00 × 10^4^ copies/ml) and negative for HBoV1 antigen in IFA, while his acute sera CMV97460 and convalescent CMV98202, collected on 30 June 2021 and 16 July 2021, respectively, were both negative in reacting with HBoV1 VP3 in IFA.

## Discussion

Since HBoV1's discovery in 2005, our laboratory has devoted deeply to accumulating data associated with its pathogenic role in pediatric patients with acute respiratory tract infection through a series of research work. We first reported in the Beijing area that infants and young children, especially those younger than 1 year, are more likely to be infected by HBoV1 (Zhao et al., 2006). By serology study, we concluded that HBoV1 had been circulating in the Beijing population for at least 10 years by 2006, and most children had been exposed to HBoV1 by the age of 7 years (Zhao et al., 2009).

The two challenges make it hard to conclude whether HBoV1 is a pathogen or only a bystander for ARTI (Schildgen et al., 2008). Cultivation of the HBoV1 remains a major challenge. It has been reported that primary air-liquid interface cell culture, polarized primary human airway epithelial (HAE) from the apical surface, provided productive infection by HBoV1 with disruption of the tight junction barrier, the loss of cilia, and hypertrophy of epithelial cells (Huang et al., 2012). In the contrary, HBoV1 causes a clear cytopathic effect resulting in damage to the three-dimensional CuFi-8 cultures, which first become smaller and finally leaky for basal media (Dijkman et al., 2009), while T84 could serve as a tool for classical virus isolation, although this has to be confirmed by further trials (Schildgen et al., 2018). However, no other common cell line or well-developed animal models support the isolation and culture of HBoV1. The second challenge is that HBoV1 DNA also persists in the lung of the convalescent population or the healthy population besides a primary infection in symptomatic patients (Christensen et al., 2019). A previous study indicated that human bocavirus DNA is present in the nuclei of infected cells, in either single or multiple copies, and appears to form concatemers (Schildgen et al., 2013). However, this does not argue against the role of HBoV1 as a true pathogen as we know that even though *P. jiroveci* is even harder to propagate in cell culture or animals than HBoV1, nobody doubts that it is a true pathogen with worldwide distribution for humans based on its closest relatives from animal models (Morris and Norris, 2012; Schildgen et al., 2014). In the study, several strategies and approaches, such as nucleic acid detection and antigen and antibody test, are referred to reveal an accurate etiological role of HBoV1.

Until now, nucleic acid detection, especially quantitative PCR (qPCR), is the most common diagnostic test for HBoV1 ARTI. In this study, there were 681 specimens (6.88%, 681/9,899) positive for HBoV1 DNA, revealing that HBoV1 is a commonly detected virus in ARTI. However, more than half (345/681, 50.66%) of the specimens showed also positive for other pathogens. It has gradually become clear that most of these so-called co-infections were co-detections because of the declining HBoV1 DNA levels from a prior ARTI that occurred weeks or months earlier (Martin et al., 2015). How do we distinguish co-infections from co-detection? So, more data from antigen or antibody tests are urged.

In this study, the monthly distribution of HBoV1 positive specimens revealed a low but consistent occurrence of HBoV1 in 2020 and a seasonal peak in the summer of 2021, which provided valuable data for understanding the prevalence of HBoV1 when a series of public health preventive measures had been widely implemented in Beijing to control the coronavirus disease-19 (COVID-19) pandemic (Zhang et al., 2021).

A commercial rapid antigen-detection assay has been developed by Bruning et al. to testify to the role of HBoV1 in ARTI (Bruning et al., 2016). Then, Kols et al. confirmed the high specificity, a higher positive predictive value, and a lower clinical sensitivity of the HBoV1 antigen detection than qPCRs (Martin et al., 2015). In this study, an HBoV1 specific antibody, anti-HBoV1 DR2, has been improved with no cross-reactivity with HBoV2 and other common viruses for antigen test. Among 47 tested, there were 37 specimens with specific positive green cells (78.72%), which illustrated the replication of HBoV1 and the pathogenic role of the disease with HBoV1 infection (Kols et al., 2019). Comparing the mean viral load of HBoV1 DNA between the antigen-positive and negative specimens revealed no significant difference (*t* = 1.119, *p* = 0.269), which indicated the inaccurate property of qPCR in building the causal relationship between the viral load and the current disease (Söderlund-Venermo et al., 2009). Otherwise, among these 37 positive specimens in the HBoV1 antigen test, 18 (48.65%, 18/37) were solely positive for HBoV1, while 19 (51.35%, 19/37) were also positive for other pathogens in the CEMP assay. For distinguishing co-infections from co-detection, an antigen test is a good choice.

The most compelling evidence in building the causal relationships between ARTI-HBoV1 is a seroconversion or a ≥4-fold increase in HBoV1-specific IgG levels. Among 4 pediatric patients, one provided positive results in nucleic acid detection, the antigen test, the seroconversion test and found a ≥4-fold increase in IgG levels. Two pediatric patients provided positive results in nucleic acid detection and the seroconversion test and found a ≥ 4-fold increase in IgG levels, while missed antigen results were shown because of the lack of cells or the full of squamous epithelial cells, revealing the defect of the antigen test. However, one pediatric patient was positive for HBoV1 DNA with negative results in the antigen test and the serology test, which suggested the inaccurate character of PCR in building the causal relationship. Due to the advantages and disadvantages of these various methods, the results combined with the nucleic acid detection, the antigen test, and the seroconversion test and a ≥4-fold increase in IgG levels in the study supported the conclusion that HBoV1 is a genuine pathogen for ARTI.

It is a pity that, although we have tried our best to collect the acute and convalescent sera from children, only 4 pairs were collected. More pairs of sera will be collected in future research. Another pity is that we only got 47 NPA samples eligible for the antigen test. For the lack of the HBoV1 specific antibody, slides obtained from 2017 to 2021 were stored at−80°C until the HBoV1 specific antibody was defined 2 months ago. Storing slides for 3–5 years is too far a long period. More slides were collected from 2020 to 2021 than that collected in 2017 and 2018 suited for antigen tests in IFA. For antigen tests, throat swabs used in the CEMP assay, a common sample type, were excluded, while specimens with more than 25 squamous epitheliums cannot be used. Altogether, we combined the nucleic acid detection, the antigen test, and the seroconversion test and found a ≥4-fold increase in IgG levels specific to HBoV1 to solidify that HBoV1 is a genuine pathogen for ARTI in pediatric patients. At the same time, our results made clear the shortcoming of every single method alone, including the inaccurate property of qPCR in building the causal relationship between the viral load and the current disease, the requirement of enough and appropriate cells in the antigen test, and the difficulty in collecting the convalescent sera from children. Therefore, combining these approaches provides a feasible way to reveal an accurate pathogenic role of HBoV1.

## Data availability statement

The datasets presented in this study can be found in online repositories. The names of the repository/repositories and accession number(s) can be found in the article/supplementary material.

## Ethics statement

The original study was approved by the Ethics Committee of the Capital Institute of Pediatrics (Approval number: SHERLLM2019013) and was a retrospective and prospective study. The ethical committee had voted that written informed consents were not required for HBoV DNA positive specimens collected in the retrospective, while those were required for HBoV DNA positive specimens collected in the prospective part. Written informed consent from the participants' legal guardian/next of kin was not required to participate in this study in accordance with the national legislation and the institutional requirements. Written informed consent was obtained from the minor(s)' legal guardian/next of kin for the publication of any potentially identifiable images or data included in this article.

## Author contributions

RD: methodology, formal analysis, and writing-original draft. KXZ: methodology and formal analysis. FW and YTZ: data curation and methodology. YS: software. DMC: investigation. RNZ, QG, SL, and DQ: resources. YQ: supervision and visualization. LQZ: project administration, writing-review and editing, conceptualization, supervision, and funding acquisition. All authors contributed to the manuscript revision, read, and approved the submitted version.

## Funding

This work was supported by grants from the Beijing Natural Science Foundation of China (No. 7192029), the National Natural Science Foundation of China (No. 82172277), and the Pediatric Medical Coordinated Development Center of the Beijing Hospitals Authority (XTZD20180505).

## Conflict of interest

The authors declare that the research was conducted in the absence of any commercial or financial relationships that could be construed as a potential conflict of interest.

## Publisher's note

All claims expressed in this article are solely those of the authors and do not necessarily represent those of their affiliated organizations, or those of the publisher, the editors and the reviewers. Any product that may be evaluated in this article, or claim that may be made by its manufacturer, is not guaranteed or endorsed by the publisher.

## Supplementary material

Supplementary Figure S1 Anti-human bocavirus 1 (anti-HBoV1) DR2 reacted with cells infected with respiratory syncytial virus (RSV) **(A)**, influenza virus (Flu) A and B **(B,C)**, human adenovirus virus (HadV) **(D)**, parainfluenza virus (PIV) 1–3 **(E–G)**, and negative control **(H)** without specific green fluorescence.

Supplementary Figure S2 Specific green fluorescent cells (positive cells) are shown in the reaction of rabbit anti-HBoV1 DR2 sera (1:40, 1:80, and 1:160) with HBoV1 VP3 expressed and clinical specimens D9466 by IFA, respectively.
